# Effect of autologous platelet leukocyte rich plasma injections on atrophied lumbar multifidus muscle in low back pain patients with monosegmental degenerative disc disease

**DOI:** 10.1051/sicotj/2016002

**Published:** 2016-03-22

**Authors:** Mohamed Hussein, Tamer Hussein

**Affiliations:** 1 Department of Orthopedics and Traumatology, Surgery New Hospital, Zagazig University Hospitals and Faculty of Medicine, Zagazig University 44519 Zagazig City, Sharkiah Egypt; 2 Department of Anesthesiology and ICU, Surgery New Hospital, Zagazig University Hospitals and Faculty of Medicine, Zagazig University 44519 Zagazig City, Sharkiah Egypt

**Keywords:** Chronic low back pain, Paraspinal muscles, Atrophied lumbar multifidus muscle, PLRP

## Abstract

*Background*: Lumbar multifidus muscle dysfunction and chronic low back pain are strongly correlated. There is no consensus regarding treatment of chronic LBP. The effect of platelet leukocyte rich plasma (PLRP) injections on atrophied lumbar multifidus (LMF) muscle and chronic low back pain has never been studied before.

*Patients and methods*: One hundred fifteen patients with chronic non-specific LBP fulfilled the inclusion criteria. Patients were treated with weekly PLRP injections for six weeks and followed up for 24 months. Primary outcome measures included Numerical Rating Scale (NRS) and Oswestry Disability Index (ODI). Secondary outcome measures included Patient Satisfaction Index (PSI), modified MacNab criteria, and lumbar MRI at 12 months follow-up.

*Results*: One hundred and four patients completed the trial. There were no serious complications. NRS significantly improved gradually from a mean of 8.8 ± 8 pre-injection to 3.45 ± 2.9 by 12 months and ODI significantly improved gradually from a mean of 36.7 ± 3.9 to 14.6 ± 12.8 by 12 months (*P* < 0.005). After reaching maximum improvement between 12 and 18 months, all outcome measures remained stable till the end of the 24 months follow-up period with statistically insignificant changes (*P* > 0.05). 87.8% (65/74) of the satisfied patients showed increased cross-sectional area and decreased fatty degeneration of LMF muscle on MRI at 12 months follow-up.

*Conclusion*: PLRP injections into atrophied lumbar multifidus muscle represent a safe, effective method for relieving chronic low back pain and disability with long-term patient satisfaction and success rate of 71.2%. We recommend the use of the lumbar PLRP injections of LMF muscle to refine the inclusion criteria of lumbar fusion to avoid failed back syndrome.

## Introduction

Approximately 70–85% of all people experience low back pain sometimes in their life [1]. The lumbar multifidus muscle has been proven clinically to be the source of low back pain and referred pain [2]. In healthy individuals, it is believed that the paraspinal muscles, especially the multifidus, play a key role in stabilization of the spine [3]. Atrophy of the paraspinal muscles occurs in chronic LBP with monosegmental degenerative disc disease [4] with reduction in cross-sectional area at L4 and/or L5 [5].

There is no consensus regarding treatment of chronic LBP [1, 6]. Paravertebral corticosteroids with or without local anesthetics infiltrations have several local and systemic deleterious side effects [7, 8]. However, Back education, physiotherapy with gym-ball exercise program, and perifacet corticosteroid injections may be effective in short-term relief of chronic LBP during the period of treatment [9]. Surgical treatment is controversial and has failed to demonstrate any beneficial effects when compared with nonsurgical treatment or placebo [10]. Moreover, surgery tends to limit motion, thus increasing stresses on adjacent motion segments. Revision lumbar fusion surgery has a clinical failure rate as high as 40% with non-union rate of 5–35% of patients [11].

There are over 1500 proteins within platelets and among them are growth factors stored in platelets as granules, which are known to play important roles in the normal healing response, including *PDGF*, *VEGF*, *TGF-B*, *bFGF*, *EGF*, *CTGF*, and *IGF-1* [12].

Leukocytes remove tissue debris and they are primarily phagocytic. They secrete growth factors (ILGF) and have a role in balancing the pro-inflammatory and anti-inflammatory aspects of healing [13].

A review of the literature revealed no clinical studies concerning PLRP injection in atrophied LMF muscle in patients with monosegmental lumbar disc degeneration. We hypothesized that PLRP injection may decrease the chronic LBP and improve disability and quality of life of those patients.

### Patients and methods

One hundred thirty-four patients who complained of chronic LBP with or without nonradicular leg pain continuously for at least three months underwent clinical examination, standardized plain radiography (lateral, oblique, and anteroposterior standing views), and MRI of the lumbar spine at the Orthopedic Department, Zagazig University Hospitals, Egypt. All the patients were assessed clinically and radiographically by two spine surgeons who were not affiliated with the study to determine the inclusion and exclusion criteria. Patients who fulfilled the inclusion criteria were treated with weekly PLRP injection for six weeks into the LMF muscle during the period from May 2012 to October 2012, and then followed up for 24 months to October 2014. All injections were performed by one orthopedic surgeon (M.H.). All the participants gave their written consent in accordance with the Helsinki Declaration [14].

#### Inclusion criteria

1. Diffuse unilateral or bilateral continuous LBP for at least three months, with or without leg pain below or above the knee.
2. Moderate (10–50%) and severe (>50%) LMF muscle atrophy in the MRI scan [15] and one-level degenerative disc disease without disc material extrusion in the canal as shown in the MRI (Figure 1).
  **Figure 1.**
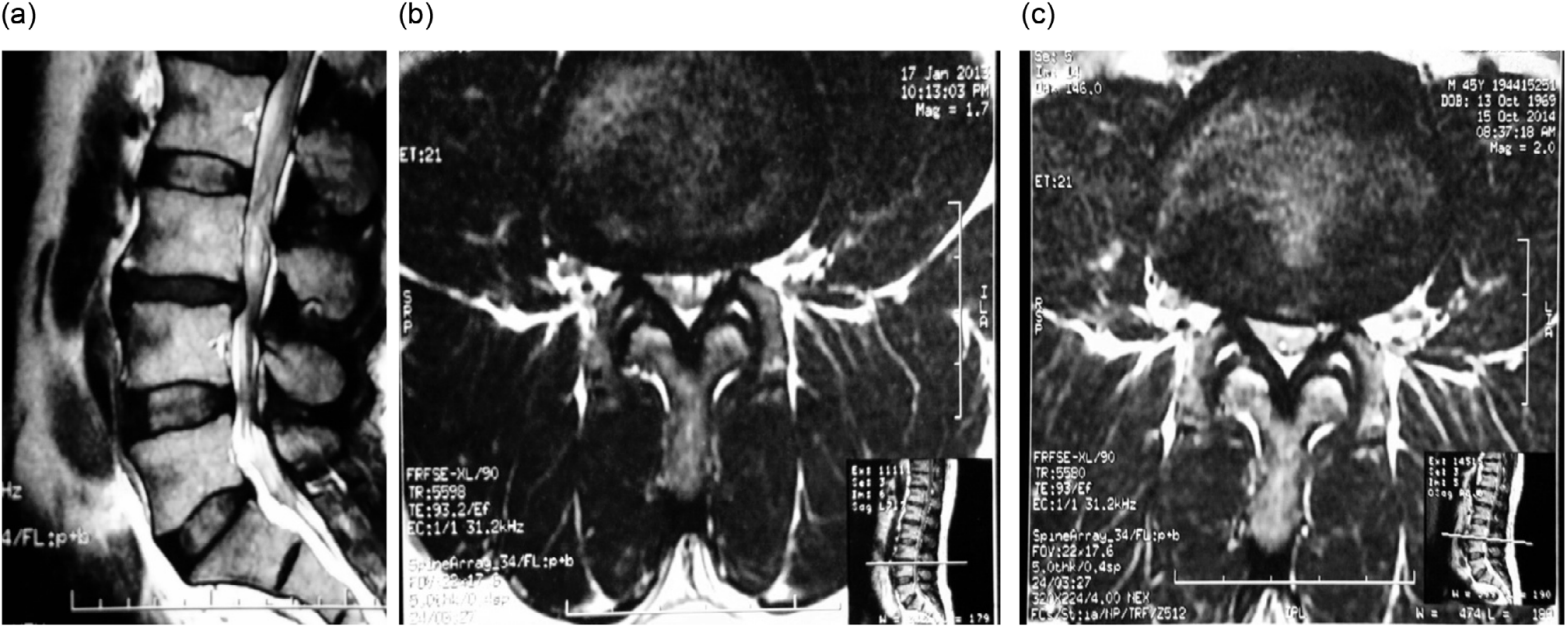
MRI lumbar spine; (a) sagittal view shows L3-4 disc degeneration, (b) axial view of the same patient shows atrophied multifidus on the same level of L3-4 disc degeneration, (c) increased cross-sectional area of LMF after 12 months of local intramuscular PLRP injection.

LMF muscle atrophy is identified by a decrease in the muscle size and deposition of fat and connective tissue, which shows a high signal intensity on the fast spin echo T2-weighted images used.

#### Exclusion criteria

1. Mild changes of less than 10% of the muscle bulk in the superiomedial part of the LMF muscle as variation of normal [15].
2. Symptomatic spinal stenosis.
3. Disc herniation (extrusion or sequestration) with clear signs of sciatica with positive straight-leg raising (SLR) test.
4. Spondylolysis, spondylolisthesis, and multisegmental degenerative disc disease on the MRI scan.
5. Scoliosis, fracture, previous spine surgery, neuromuscular disease of the trunk, malignancy, infection, and pregnancy.

Thus, after exclusion; the inclusion sample size used in this study consisted of 115 patients and after removing 11 patients who did not complete the injection protocol or the 2-years follow-up period, the final sample size consisted of 104 patients (90.4% of the included patients). Fifty-six men and 48 women with a mean age of 30.8 ± 9.21 years (range 17–55). The mean duration of symptoms was 6.3 ± 2.4 months.

### Evaluation

Primary outcome measures included (NRS) (range 0–10) [16] for intensity of pain and ODI [17] – version 2.0 (the sex question [Section 8] is unacceptable in our community, therefore it was removed from the questionnaire). The total possible score became 45. The final score is calculated and presented as a percentage (0% represents no pain and disability and 100% represents the worst possible pain and disability). Secondary outcome measures included complication rate, PSI [18], modified MacNab criteria [19], and lumbar MRI done at 12 months follow-up.

### Platelet leukocyte rich plasma preparation

Fifty milliliters of venous blood sample was collected in sterile tubes containing ACD solution as anticoagulant. The samples were centrifuged twice: the first at 1500 rpm for 15 min. which separates erythrocytes. The second centrifugation at 3000 rpm for 20 min to concentrate plasma components to produce a unit of 5 mL of PLRP. One milliliter of the prepared PRP was left for a platelet count, which was done by an automated hemocytometer Cell-Dyn 3700 (Abbott Labs, Dallas, TX, USA). Platelet count ranged 0.8–1.2 million/mL. with a mean increase of 4–6-fold of the whole blood values. The prepared amount of PLRP was mixed with 10% calcium chloride for activation of platelets immediately before injection (Ca2 = 0.22 m Eq × dose). The amount of PRP prepared is 5 mL (10%) of the amount of the whole blood withdrawn from the patient, following the literature given in references [20–22].

### Treatment procedure and follow-up

After sterile dressing of the skin with Betadine (povidone-iodine), the area was partially anesthetized by spraying ethyl chloride onto the skin. Surgical gloves were worn by the physician during the injections. Injections were performed using a 21-g spinal needle, utilizing the posterior paramedian portal 1.5 cm laterally from the midline with the patient lying prone over the examination table. Under fluoroscopic guidance the needle is directed vertically (0°) in the axial plane and 5–10° caudally in the sagittal plane until the tip of the needle hits the lamina of the upper vertebra of the degenerated lumbar motion segment to reach the deep fibers of the LMF muscle. Fix the syringe containing the 5 mL PLRP to the spinal needle and withdraw the tip of the needle 1–2 mm. Then, inject slowly (2.5 mL per side). During the treatment period, patients are instructed to stop the use of NSAIDs and use cold foments on the lumbar region for pain relief. Patients were advised to walk daily for at least 30 min and remain as active as possible.

### Follow-up

Follow-up data were obtained from clinic follow-up visits and telephone calls by two independent physicians; before PLRP injections (115 patients), after PLRP injections at day 1 (113 patients in outpatient clinic), 6 months (109 patients: 73 in outpatient clinic and 36 by phone calls), 1 year (105 patients: 67 in outpatient clinic (OPC), 38 by phone calls), and at the final follow-up visit 2 years (104 patients: 67 in OPC, 37 by phone calls) (90.4%). The remaining patients were lost for the following reasons: four treatment-unrelated deaths and seven patients who did not respond to telephone calls were excluded from the analysis. Eight patients who underwent intervertebral fusion during the 24 months follow-up period were considered as failure and were included in the analysis.

### Statistical analysis

All statistical analyses were carried out using the SPSS (Statistical Package of Social Sciences, Chicago, IL, USA) for Windows software program version 17.0. *P* value of less than 0.05 was considered statistically significant. The results were expressed as mean ± sd. Paired *t* test and the One-Way ANOVA were used to test for significant differences between baseline band and various follow-up measurements. Chi-square test was used to test the differences between the two groups in terms of categorical data. The General Linear Model (GLM) was used as multivariate analysis to assess the influence of patient characteristics over months of benefit with sex and degree of LMF muscle atrophy as a fixed effect and age and BMI as covariates. Pearson correlation analysis was used to find the correlation between the pre-injection and post-injection measurements for pain and disability.

## Results

There were no serious complications, such as nerve-root injury, multifidus muscle abscess, cauda equina syndrome, spondylodiscitis, or thrombosis. All the patients experienced injection site pain during injection that may last for one or two days. They were prescribed paracetamol or acetaminophen and a muscle relaxant after each PLRP injection to control pain and muscle spasm of the initial inflammatory phase. One hundred and four patients were included in the analysis, 30 patients (28.8%) did not show any improvement (including the eight patients who underwent lumbar fusion) and the remaining 74 patients (71.2%) still had good results without worsening over time at the 24 months follow-up evaluation. The mean duration of the beneficial effects was 18 ± 6 months.

The mean NRS back pain score significantly decreased from 8.81 ± 0.86 pre-injection, to 3.5 ± 2.92 by the end of the 24 months follow-up period with a mean difference of 5.31 ± 2.90 (*P* > 0.001). The mean ODI score significantly decreased from 36.74 ± 3.95 pre-injection, to 14.65 ± 12.8 by the end of the 24 months follow-up period with a mean difference of 22.09 ± 12.7 (*P* > 0.001) (Table 1).

**Table 1.** Mean pre-injection and post-injection NRS (back) and ODI for lumbar multifidus muscle PLRP injection through the follow-up period.

Outcome	Pre-inj. scores	Day 1	6 months	12 months	18 months	24 months	Difference in group means (95% CI)
NRS-back pain	8.8 ± (0.86)	7.2 ± (1.1)	4.57 ± (2.48)	3.45 ± (2.94)^[1]^	3.48 ± (2.93)^[2]^	3.5 ± (2.92)	5.31 ± (2.90)
ODI	73.48% (3.95)	63.1% (4.5)	29.44% (12.8)^[3]^	29.28% (12.8)^[4]^	29.26% (12.8)^[5]^	29.3% (12.8)	44.18% (12.7)

Values are mean standard deviation (SD), NRS = Numerical Rating Scale (0–10), ODI = Oswestry Disability Index, CI = Confidence Interval, the mean ODI score is multiplied by two to give the mean disability score which is expressed in the table. Difference in group means is expressed as the difference between pre-injection and post-injection values at the end of follow-up period. The mean ODI and (NRS) back scores for lumbar multifidus PLRP injection showed significant improvement at the end of the 2-years follow-up period, the mean ODI and (NRS) back scores showed statistically insignificant changes and remained low without significant increase again after initial significant improvement that started at the day-1 follow-up that reached maximum improvement by 6–12 months follow-up period post-injection. *P* (paired *t* test) < 0.001 *[1] *P* = 0.181, [2] *P* = 0.32, [3] *P* = 0.083, [4] *P* = 0.741, [5] *P* = 0.32.

The General Linear Model (GLM) showed that the months of benefit were influenced by the degree of LMF muscle atrophy (eta-squared *η*^2^ = 0.567, *P* = 0.00), and not influenced by BMI (*η*^2^ = 0.003, *P* = 0.564), age (*η*^2^ = 0.005, *P* = 467), or sex (*η*^2^ = 0.023, *P* = 0.13) (Table 2). Each term in the model (age, sex, BMI, and LMF muscle atrophy), plus the model as a whole, is tested for its ability to account for variation in the dependent variable (which is the duration of benefit). The significance value for each term, except LMF muscle atrophy, is more than 0.05. Therefore each term, except LMF muscle atrophy, is statistically insignificant (i.e. has no effect on the duration of benefit).

**Table 2.** Explains the practical significance of each variable on the duration of benefit.

Tests of between-subjects effects						
Dependent variable: month of benefit						
Source	Type III sum of squares	*df*	Mean square	*F*	Sig.	Partial eta squared
Corrected model	2477.648^a^	5	495.530	48.066	.000	.710
Intercept	154.738	1	154.738	15.010	.000	.133
Age	5.280	1	5.280	.512	.476	.005
BMI	3.460	1	3.460	.336	.564	.003
MF atrophy	1323.191	1	1323.191	128.349	.000	.567
sex	24.014	1	24.014	2.329	.130	.023
MF atrophy × sex	10.914	1	10.914	1.059	.306	.011
Error	1010.313	98	10.309			
Total	16028.000	104				
Corrected total	3487.962	103				

a *R*^2^ = .710 (adjusted *R*^2^ = .696). Larger values of partial (*η*^2^ = eta squared) indicate a greater amount of variation accounted for by the model term, to a maximum of 1. Here the individual term (degree of LMF atrophy) is statistically significant and has great effect on the value of months of benefit.

According to Pearson correlation analysis; there is a strong correlation between the pre-injection and day-1 post-injection measurements for NRS which is statistically significant, indicating that pain level was lower in all patients (i.e. consistent decrease), but for the remaining follow-up measurements, the correlations were weak and they were statistically insignificant indicating that the change was inconsistent across all patients (i.e. several lowered their levels of pain, but several others either did not change or increased their levels). There is a strong correlation between the pre-injection and all the post-injection measurements for ODI score which is statistically significant, indicating that disability level was lower in all patients (i.e. consistent decrease). However, for the day-1 post-injection measurements, the correlation was weak and it was statistically insignificant indicating that the change was inconsistent across all patients. This may be explained by the time needed by the atrophied LMF muscle to regain its strength.

**Figure 2.**
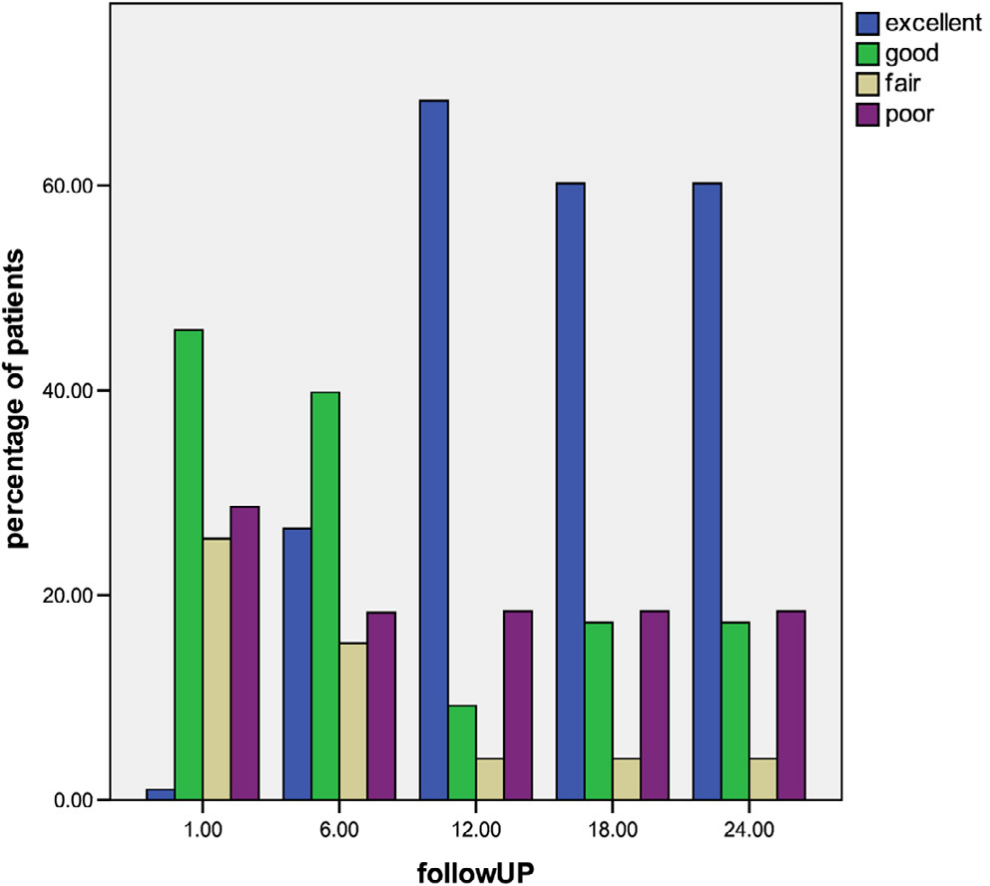
During the first 12 months of the follow-up period the percentage of patients with (excellent) and (good) outcome increased reaching maximum at 12 months follow-up, whereas the percentage of patients with (fair) and (poor) outcome decreased and then remained stable until the end of follow-up.

According to modified MacNab criteria, by 18 months post-injection 60.2% had excellent outcomes, 17.3% good, 4.0% fair, and 18.4% poor; these results remained unchanged throughout the 24 months follow-up period. If the excellent and good categories were regarded as success and fair and poor as failures, then the total success rate was 71.2% and failure rate was 28.8% (Figure 2).

**Figure 3.**
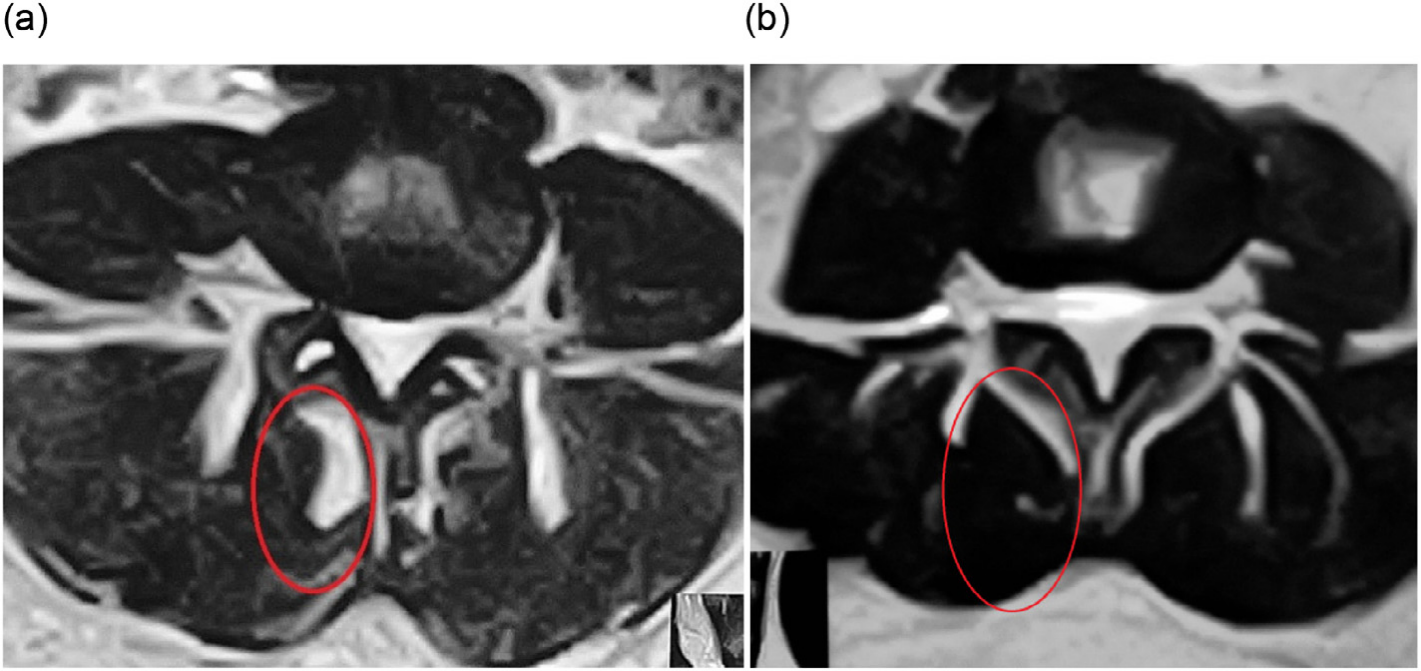
(a) Axial view of lumbar spine MRI shows severe multifidus atrophy, fatty degeneration (high signal intensity) is nearly 50% (red circle), (b) axial view of the same patient after one year post-injection of PLRP into atrophied lumbar multifidus with decreased fatty degeneration.

**Figure 4.**
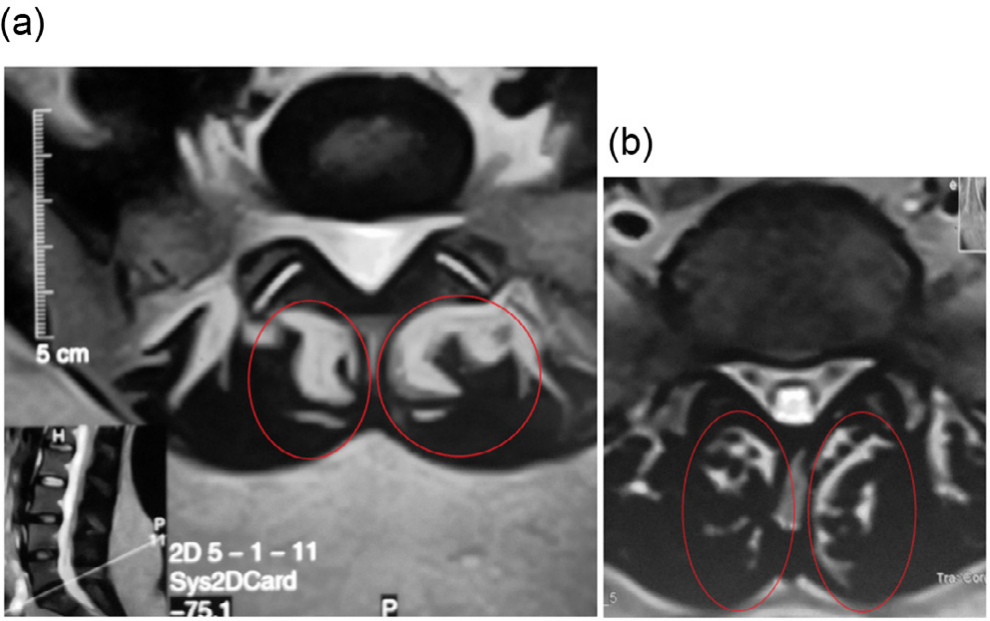
(a) Axial view of lumbar spine MRI shows severe multifidus atrophy, fatty degeneration (high signal intensity) is more than 50% (red circle), (b) axial view of the same patient after 15 months post-injection of PLRP into atrophied lumbar multifidus with decreased fatty degeneration.

According to PSI, 71.2% (74/104) of the patients showed complete satisfaction with the procedure and outcome, and would undergo the injection again for the same condition. While 28.8% (30/104) of the patients were unsatisfied. 87.8% (65/74) of the satisfied patients showed improvement on post-injection MRI done at 12 months of follow-up in the form of increased cross-sectional area and/or decrease in the fat and fibrous tissue content (decrease in high signal intensity on T2-W axial cuts) (Figures 3 and 4).

## Discussion

Our study is the first study that clinically correlates persistent LBP to atrophied LMF muscles by evaluation of the clinical response of LBP patients to PLRP injections of the atrophied LMF muscle. Our preliminary results indicate that chronic LBP is not always due to disc degeneration and instability alone. Also, it proves the role of LMF muscle atrophy as a cause of chronic LBP with or without leg pain. In 2014 Kuittinen et al. [23] did not find any correlation between objective quantitative MRI measures and patient symptoms in degenerative spinal canal stenosis; they suggested other underlying pathobiological mechanisms yet to be discovered.

We suggest a self-sustained vicious cycle which starts with an injury to either the disc, the LMF muscle, or the facet joint. It can lead to further injury to the other two components. Some imaging studies found correlation between chronic LBP and atrophied LMF muscle accompanied by monosegmental degenerative disc disease (two-components) [4, 24]. As long as the injury is limited to one component, the lumbar spine can withstand its loads without pain or disability. Symptom-free people (20–30%) often have demonstrable lumbar disc degeneration or herniation on MRI [25, 26].

We observed a short-term reduction in pain after PLRP injections. Platelet concentrates had an analgesic effect [20, 27]. This phenomenon was explained by the presence of large amount of serotonin released from the dense granules of the activated concentrated platelets at the injection site [28]. Overall, the improvement of pain, disability, success rate and patient satisfaction were statistically significant and reached their maximum between 12 and 18 months, then remained stable throughout the follow-up period. This delayed clinical improvement can be explained by time needed by paravertebral neuromuscular tissues to regain its biotensegrity especially the LMF muscle, but proving that it needs further research.

A limitation in our study was the absence of a control group because as mentioned above, there is no consensus regarding treatment of chronic LBP [1, 6]. Paravertebral corticosteroids with or without local anesthetics infiltrations are generally performed in the vicinity of nerve roots, even facet infiltration is also a type of nerve-root block, as it is intended to block the posterior branch that emerges directly from the spinal nerve [6–9]. This would create a state of (iatrogenic denervation) increasing the LMF muscle atrophy as denervation leads to atrophy and increased intramuscular fat [29]. Paravertebral corticosteroid infiltrations suppress inflammatory mediators and protein synthesis and suppress hypothalamic-pituitary-adrenocortical axis and immunity leading to severe infection [7]. So, we were reluctant to expose the patients in this study to the possibility of corticosteroid side effects for a return of temporary pain relief. We considered this to be unethical.

We used PLRP concentrate of physiological proportions that included concentrated platelets and leukocytes instead of PRP. If platelets are not suspended with biologic levels of other constituents of plasma such as leukocytes, cytokines, and fibrin (the Matrix), the platelet concentrate is either not effective or less effective [21]. We had no complications particularly local infection and this may be attributed to the concentrated leukocytes. The density of a leukocyte is approximately the same as a platelet and as a result the procedure also concentrates leukocytes to almost a 10-fold increase. Leukocytes contain some growth factors such as the PDGF-AB that originates from leukocytes in addition to ILGF. Leukocytes have also been shown to produce combinations of proteases and SLPI which inhibits excessive protease activity that leads to destruction of soft tissue and inhibits transition to the proliferative phase of healing [30]. Serhan [31] discovered lipoxins which are proteins secreted by human leukocytes and (LXA4) are formed by 12-lipoxygenase of platelets that depend upon neutrophils for its precursor (LTA_4_). El-Sharkawy and colleagues [32] studied platelet secretions and their effect on macrophage cultures, concluding that PLRP in the 4–6-fold range increases RANTES and Lipoxin A4 (LXA4) which suppresses cytokine release leading to anti-inflammatory effects. Until now, no undesirable inflammatory reactions have been observed with leukocyte rich PRP, even in immune-sensitive applications [13].

We recommend the use of the PLRP injections of atrophied LMF muscle as a safe method that may decrease the chronic LBP and improve disability and quality of life of patients with monosegmental lumbar disc degeneration. We also recommend it as a method to refine the inclusion criteria of lumbar fusion to avoid failed back syndrome.

## Abbreviations

ACD: Acid citrate dextrose
bFGF: Basic fibroblast growth factor
BMI: Body mass index
CTGF: Connective tissue growth factor
EGF: Epidermal growth factor
GLM: General Linear Model
IGF-1: Insulin-like growth factor-1
LBP: Low Back Pain
LMF: Lumbar Multifidus
LXA4: Lipoxin A4
MD: Mean difference
NRS: Numerical Rating Scale
NSAIDs: Nonsteroidal anti-inflammatory drugs
ODI: Oswestry Disability Index
OPC: Outpatient clinic
PDGF: Platelet derived growth factor
PLRP: Platelet leukocyte rich plasma
PRP: Platelet rich plasma
RANTES: Regulated on activation, normal T cell expressed and secreted
SLPI: Secretory leukocyte protease inhibitor
TGF-B: Transforming growth factor beta
VEGF: Vascular endothelial growth factor

## Conflict of interest

No external funding was received in support of the present study. No benefits in any form have been or will be received from a commercial party related directly or indirectly to the subject of this manuscript.

M.H. and T.H. certifiy that they have no financial conflict of interest (e.g., consultancies, stock ownership, equity interest, patent/licensing arrangements, etc.) in connection with this article.
